# Preimmune Control of the Variance of TCR CDR-B3: Insights Gained From Germline Replacement of a TCR Dβ Gene Segment With an Ig D_*H*_ Gene Segment

**DOI:** 10.3389/fimmu.2020.02079

**Published:** 2020-09-11

**Authors:** Mohamed Khass, Michael Levinson, Robert L. Schelonka, Pratibha Kapoor, Peter D. Burrows, Harry W. Schroeder

**Affiliations:** ^1^Division of Clinical Immunology and Rheumatology, Department of Medicine, The University of Alabama at Birmingham, Birmingham, AL, United States; ^2^Division of Genetic Engineering and Biotechnology, National Research Center, Cairo, Egypt; ^3^Division of Neonatology, Department of Pediatrics, Oregon Health Science Center, Portland, OR, United States; ^4^Department of Microbiology, University of Alabama at Birmingham, Birmingham, AL, United States; ^5^Division of Clinical Immunology and Rheumatology, Department of Medicine, Microbiology, and Genetics, The University of Alabama at Birmingham, Birmingham, AL, United States

**Keywords:** germline, T cell receptor, gene segment, immunoglobulin, D gene segment

## Abstract

We have previously shown that the sequence of the immunoglobulin diversity gene segment (D_*H*_) helps dictate the structure and composition of complementarity determining region 3 of the immunoglobulin heavy chain (CDR-H3). In order to test the role of germline D sequence on the diversity of the preimmune TCRβ repertoire of T cells, we generated a mouse with a mutant TCRβ DJC locus wherein the Dβ2-Jβ2 gene segment cluster was deleted and the remaining diversity gene segment, Dβ1 (IMGT:TRDB1), was replaced with DSP2.3 (IMGT:IGHD2-02), a commonly used B cell immunoglobulin D_*H*_ gene segment. Crystallographic studies have shown that the length and thus structure of TCR CDR-B3 places amino acids at the tip of CDR-B3 in a position to directly interact with peptide bound to an MHC molecule. The length distribution of complementarity determining region 3 of the T cell receptor beta chain (CDR-B3) has been proposed to be restricted largely by MHC-specific selection, disfavoring CDR-B3 that are too long or too short. Here we show that the mechanism of control of CDR-B3 length depends on the Dβ sequence, which in turn dictates exonucleolytic nibbling. By contrast, the extent of N addition and the variance of created CDR3 lengths are regulated by the cell of origin, the thymocyte. We found that the sequence of the D and control of N addition collaborate to bias the distribution of CDR-B3 lengths in the pre-immune TCR repertoire and to focus the diversity provided by N addition and the sequence of the D on that portion of CDR-B3 that is most likely to interact with the peptide that is bound to the presenting MHC.

## Introduction

V(D)J rearrangement has been calculated to yield potential repertoires of more than 10^16^ different T cell receptor (TCR) or immunoglobulin (Ig) antigen binding sites (1, 2). A fundamental issue is the extent to which diversity is random or directed. The former would imply that diversification of the repertoire is purely a matter of chance. The latter would suggest that diversification takes place under germline control in order to optimize the creation of a functional repertoire and minimize autoreactive clones.

A major component of antigen receptor diversity comes from the inclusion of a diversity (D) gene segment into the rearrangement process. In B cells, D gene segments contribute to Ig heavy (H) chain diversity and in αβ T cells they contribute to TCRβ diversity. In both antigen receptors, amino acids encoded by the D are positioned at the center of complementarity determining region 3 (CDR-H3 for the immunoglobulin H chain and CDR-B3 for the T cell receptor beta chain), which is the direct product of V(D)J rearrangement (3). In both Ig and TCR, D gene segment-encoded amino acids within CDR3 commonly contribute directly to the recognition and binding of cognate antigens. The inclusion of a D gene segment also allows two rounds of junctional diversification during VDJ rearrangement. The somatic mechanisms of CDR3 junctional diversification include terminal exonucleolytic “nibbling,” P junction extension and N nucleotide addition.

Although potential CDR3 diversity is astronomic, we have previously shown that there are constraints on the structures of the immunoglobulin CDR-H3 repertoire that can be detected through analysis of as few as ten to twenty sequences, not thousands or millions. For example, in progenitor B cells, constraints on germline D_*H*_ sequence can heavily influence the structure and composition of immunoglobulin H chain (CDR-H3) (4). Thus, constraints on D_*H*_ germline content represent one mechanism by which the structural diversity of the repertoire can be directed.

In order to further test the role of germline D sequence on the shape of the preimmune CDR3 repertoire, we turned to the TCRβ locus and created a mouse with a mutant TCRβ DJC locus wherein the Dβ2-Jβ2 gene segment cluster had been deleted (Dβ2ko) and the remaining Dβ1 gene segment [ImMunoGeneTics (IMGT) database (5) (IMGT: TRBD1)] replaced with a commonly used D_*H*_ gene segment DSP2.3 [IMGT: IGHD2-7(BALB/c)].

We found that the mechanism of control of CDR-B3 length, which is important for optimal MHC:peptide interactions, depends on the Dβ sequence, which in turn dictates exonucleolytic nibbling. Conversely, the extent of N addition and the variance of created CDR3 lengths are regulated by the cell of origin, the thymocyte.

## Materials and Methods

### Generation of Targeted ES Cells and the DβYTL Mouse

Plasmids containing the germline C57BL/7 Dβ1 and Jβ1 loci were the kind gift of Dr. Barry Sleckman. The targeting construct was generated using a pLNtk targeting vector containing a *Sal*I–loxP-Neo^*r*^-loxP–*Xho*I-TK cassette (Supplementary Figure S1). A 4.4 kb *Kpn*I*-Sac*II 3′ homology arm containing the Jβ1 gene segments was subcloned into the *Xho*I site by blunt-end ligation.

A plasmid (BSSK5′M) containing *D*β*1* was used as a substrate for PCR directed replacement of TCRβ *D*β*1* by IgH *DSP2.3*. Overlapping 64 base pair primers containing the sequence of DSP2.3 in place of Dβ1 were generated. These were 5′ tgtataaagctgtaa cattgtg TCTACTATGGTTACGAC cacggtg attcaattctatgggaag 3′ and 5′ cttcccatagaattgaat caccgtg GTCGTAACCATAGTAGA cacaatg ttacagctttataca 3′. The sequence of the D is in caps and the heptamers are separated by spaces from the rest of the sequence. Each of these was individually paired with a forward primer (5′ ataacctctgaggacgcacagccttaggg 3′) upstream of a *Bsu*36I site and a reverse primer (5′ acgactcactatagggcgaattgggtaccg 3′) downstream of a *Hin*dIII site. The overlapping PCR products were then annealed and PCR amplification was performed with the upstream of *Bsu*36I and downstream of *Hin*dIII primers alone. The resulting PCR amplified product was cut with *Bsu*36I and *Hin*dIII, and back cloned into the BSSK5′M plasmid, thus replacing *D*β*1* with *DSP2.3.*

A 2.6 kb *Not*I-*Cla*I 5′ homology arm was cut from the BSSK5′M plasmid and subcloned by blunt-end ligation into a *Sal*I upstream of the first loxP site in the pLNtk + 3′ homology arm plasmid. The resulting 14.9 kb targeting vector was linearized with *Pvu*I and electroporated into 129 derived DJβ2^–/–^ (Dβ2ko) mouse ES cells (6, 7). Briefly, 1 × 10^7^ ES cells were electroporated with 25 μg of linearized vector DNA in a 0.4 cm cuvette at 240 V and 500 μF (Bio-Rad Gene Pulsar, Bio-Rad Laboratories, Hercules, CA). Individual ES cell clones were selected with 200 μg/mL G418 (positive selection) and 2 μM Ganciclovir (negative selection) from 24 h after electroporation for a total of 2 weeks. The transfection efficiency was 3%.

ES cell clones with homologous recombination were identified by long PCR using LA Taq DNA polymerase (Takara Bio USA, Mountain View, CA, United States). The PCR program used was (1) denaturation at 94°C for 1 min, (2) 94°C for 20 sec, 68°C for 7 min for 31 cycles, (3) 68°C for 10 min, and (4) hold at 4°C. The primers used to identify the correct 5’ end of the recombinant were a 5′ primer from the mouse TCRDβ1 region (5′ gtgagtccatcattgctagggaaaggggttgagtg 3′) and a 3′ primer from the Neo loxP region of the targeting vector (5′ gagcccagaaagcgaaggaacaaagctgctattgg 3′). The primers used to identify the correct 3′ end of the recombinant were a 5′ primer from Neo loxP region (5′ acgggggtgggggtggggtgggattagataaatgc 3′) and a 3′ primer from mouse TCRDβ1 region (5′ ccatggaactgcacttggcagcggaagtggttgcg 3′).

The TCRβ DJC locus resulting from this manipulation contained the original germline Dβ1 recombination signal sequences that now flanked immunoglobulin D_*H*_ DSP2.3 in place of Dβ1. It contained the Jβ1 and Cβ1 locus in its entirety as well as the Cβ2 constant domain but lacked Dβ2 and Jβ2 sequences. We termed this new D_*H*_ substituted TCR locus DβYTL, which refers to the central amino acids in each of its three reading frames (i.e., tyrosine, threonine, and leucine). For the purposes of this manuscript, we renamed the original DJβ2^–/–^ gene targeted locus Dβ2ko to emphasize the deletion of Dβ2.

Two original Dβ2ko ES cells and two independently derived DβYTL ES cell clones were independently microinjected into C57BL6/J blastocysts. The resulting chimeric mice were bred to wild type C57BL6/J mice. The agouti offspring were genotyped by tail DNA PCR analysis to assess germline transmission of the DβYTL or Dβ2ko TCR alleles. Homozygous DβYTL mice were bred to transgenic mice expressing the Cre recombinase from the CMV promoter to delete the LoxP-flanked Neo^*r*^ gene during early embryogenesis (Cre mice were obtained from Jackson Laboratories). Deletion of the Neo gene in the offspring was confirmed by PCR using Cre3 (5′gaatttactgaccgtacac3 ′) and Cre4 (5′catcgccatcttccagcag3 ′) primers. The homozygous progeny harboring mutant DβYTL or Dβ2ko TCR alleles were backcrossed with wild type C57BL6/J mice for 24 generations. All animal experiments were approved by the University of Alabama at Birmingham (UAB) Institutional Animal Care and Use Committee. The UAB Animal Care and Use Program is fully accredited by Association for Assessment and Accreditation of Laboratory Animal Care International.

### Flow Cytometric Analysis and Cell Sorting

For wild type (WT), DβYTL and Dβ2ko mice, single cell suspensions were prepared from the thymus of two mice each. Red blood cells were removed with RBC lysing solution (1 mM KHCO_3_, 0.15 M NH_4_Cl, and 0.1 mM Na_2_EDTA). Cells were washed twice and resuspended in a master-mix of staining buffer containing optimal concentrations of monoclonal antibody reagents. Samples were sorted with a FACS Aria (Becton Dickinson). Double negative thymic cells were stained with PE-Cy7-CD25 (BD Cat #552880), APC-CD44 (BD Cat #559250), biotinylated-CD28 (BD Cat #553296) (developed secondarily with streptavidin), and a lineage stain [PE-CD3 (BD Cat #555275), monoclonal PE-CD4 (BD Cat #553049), PE-CD8α (BD Cat #553033), PE-B220 (BD Cat #561878), PE-CD11b (BD Cat #553311), PE-NK1.1 (BD Cat#553165)] to remove mature T, B, and NK cells. The cells were stained with propidium iodide (PI) to identify/sort live cells. DN2 cells were defined as CD44^+^ and CD25^+^ (Supplementary Figure S2).

### RNA, RT-PCR, DNA Cloning, and Sequencing

Total RNA was prepared from 1 × 10^4^ to 2 × 10^4^ cells of each individual subset, sorted directly into RLT lysing buffer using a QIAGEN RNeasy mini-kit. RNA was used to synthesize cDNA using the QIAGEN RT-PCR Kit and the manufacturer’s recommended protocol under the following conditions: 95°C denaturation for 2 min; 30 cycles of 94°C for 1 min, 60°C for 1 min, and 72°C for 1 min; and a final 72°C extension for 10 min. The reaction buffer contained 100 mM Tris-HCl, pH 8.8, 15 mM MgCl_2_, and 750 mM KCl. Primers used were TCRB13-1 (5’- tgctggcaaccttcgaatagga-3’) and TCRBC1 (5’- tgagaaatgtgactccaccca-3’). PCR products were cloned (TOPO-TA Cloning Kit; Invitrogen) and sequenced using the primer TCRB13-1 on an ABI 3730 sequencer.

### Sequence Analysis of CDR-H3 and CDR-β3

Eight of the thirteen D_*H*_ gene segments in BALB/c mice and six of the ten D_*H*_ in C57BL/6 mice belong to the DSP family. Due to the extensive sequence similarity among these gene segments, it is often difficult to determine exactly which DSP germline gene segment contributed to an individual CDR-H3. Thus, we grouped all of the sequences of CDR-H3 that had identifiable DSP family sequence into wild type controls.

**FIGURE 1:**
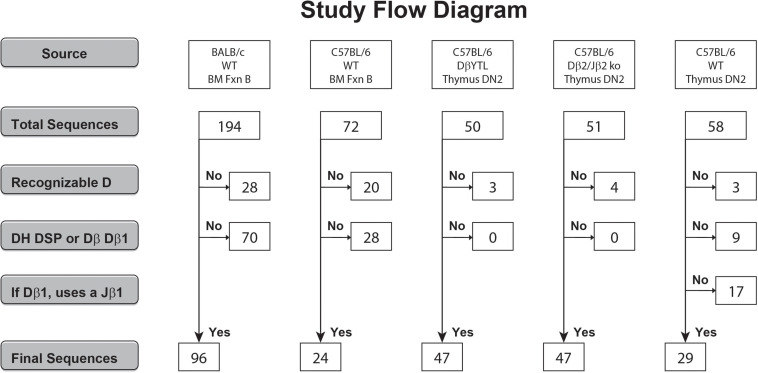
Flow diagram of the derivation and the numbers of the DSP- and Dβ1-containing CDR3 sequences analyzed. Sequences that did not meet individual criteria were discarded into the “No” pool. Sequences that met all the individual criteria were pooled into the “Yes” pool.

194 V_*H*_7183 CDR-H3 sequences from BALB/c Hardy Fraction B [CD19^+^CD43^+^Bp-1^–^IgM^–^] (8) and 72 V_*H*_7183CDR-H3 sequences from the C57BL/6 Fraction B equivalent [B220^+^ cKit^+^,CD25^–^, BP-1^–^] (9) proB cells were previously published and analyzed as a whole (4). Of these there were 96 BALB/c sequences and 24 C57BL/6 sequences that contained an identifiable member of the DSP gene segment family (Figure 1 and Supplementary Table S1).

Gene segments were assigned according to published germline sequences for the TCR β gene segments as listed in the ImMunoGeneTics database (5). The CDR3 of the TCRβ chain was defined to include those residues located between the conserved cysteine (C104) of FR3 and the conserved phenylalanine (F118) of FR4 (10). These TCRβ sequences obtained from C57BL/6 DβYTL, Dβ2ko, and wild type DN2 thymocytes were compared to the Ig CDR-H3 sequences. In total, 47 of 50, 47 of 51, and 29 of 58 thymocyte CDR-B3 sequences from the three respective mouse strains contained identifiable D_*H*_ DSP2.3 or TCR Dβ1-Jβ1 sequences (Supplementary Table S1).

### Statistical Analysis

Statistical analysis was performed with JMP version 14 (SAS Institute) or GraphPad Prism 8 version 8 (GraphPad Software, San Diego, CA, United States). Population means were analyzed using one-way analysis of variance (ANOVA) test. Variance was assessed with the O’Brien Test for Homogeneity of Variance. Categorical comparisons were performed with Fisher’s exact test.

## Results

In order to test the effect of changing the sequence of a D gene segment on VDJβ rearrangement and N addition, we replaced the Dβ1 gene segment with DSP2.3, a commonly used Ig D_*H*_ gene segment, to create a new TCRβ allele we termed DβYTL, which refers to the central amino acids in each of its three reading frames (i.e., tyrosine, threonine, and leucine). To simplify the analysis, we introduced this gene substitution mutation into an ES cell that had previously undergone a gene targeted deletion of the Dβ2-Jβ2 gene segment cluster (Dβ2ko). We chose to utilize a sequence of a member of the DSP D_*H*_ family (Table 1 and Supplementary Table S2) because together the members of this family make up a majority of functional D_*H*_ sequences in both BALB/c and C57BL/6 mice, the DSP2.3 sequence is found in the germlines of both strains and, unlike the case with many other D_*H*_ segments, none of the three D_*H*_ DSP2.3 reading frames rearranged by deletion contain a termination codon (11).

**TABLE 1:** Germline V, D, and J CDR3 contributing sequences pertinent to this study.

**Strain**	**IMGT**	**Common**	**Terminus**
BALB/c	IGHV5-01	VH81X	GCAAGACA
	IGHV5-02	VHD6.96	……G.
	IGHV5-06	VH3:3.39	……..
	IGHV5-14	7183.14	……..
	IGHV5-05	VH50.1	……..
	IGHV5-09	7183.9	……..
	IGHV5-04	VH37.1	……..
		7183.9T	……..
	IGHV5-16	98-3G	……G.
	IGHV5-03	VH283	G…..T.
	IGHV5-10	7183.1O	……..
	IGHV5-08	VH10-19	A…..G.
	IGHV5-15	68-5N	……..
	IGHV5-17	69-1	A…..G.
	IGHV5-13	7183.13	……..
	IGHV5-12	57-1M	……G.
	IGHV5-11	VH7183.11	…..GG
C57BL/6	IGHV5-17		……
	IGHV5-16		……G.
	IGHV5-15		……..
	IGHV5-14		……..
	IGHV5-12		……..
	IGHV5-09		……..
	IGHV5-06		……..
	IGHV5-04		……G
	IGHV5-02		……..
	TRBV13-1		..C..CAGTGATG
Strain	IMGT	Common	D sequence
BALB/c	D2-02*01	DSP2.03	TCTACTATGGTTACGAC
	D2-04*01	DSP2.02	………A…….
	D2-02*01	DSP2.04	……………..
	D2-01*01	DSP2.05	………..A..T..
	D2-10*01	DSP2.07	C……….A..T..
	D2-11*01	DSP2.08	C…G……A..T..
	D2-03*01	DSP2.09A	….TG……..T..
	D2-14*01	DSP2.11	C…….A.G……
C57BL/6	D2-03*01	DSP2.09A	….TG……..T..
	D2-04*01	DSP2.02	………A…….
	D2-05*01	DSP2.x	C…….A..A..T..
	D2-06*01	DSP2.x	C…….A..A..T..
	D2-07*01	DSP2.03	……………..
	D2-08*01	DSP2.05	………..A..T..
	TRDB1*01	Dβ1	GGGACAGGG.G.
Strain	IMGT	Common	Terminus
BALB/c	IGHJ1*01	JH1	CTACTGGTACTTCGATGTC
	IGHJ2*01	JH2	AC…..T..CTA.
	IGHJ3*01	JH3	CCTGG..T.C.TA.
	IGHJ4*01	JH4	AT….ATGCTA.G..CTA.
C57BL/6	IGHJ1*03	JH1	……………….
	IGHJ2*01	JH2	AC…..T..CTA.
	IGHJ3*01	JH3	CCTGG..T.C.TA.
	IGHJ4*01	JH4	AT….ATGCTA.G..CTA.
	TRBJ1-1*01	Jβ1	CAAACACAGAA.TCT..
	TRBJ2-2*01	Jβ2	CAAACTC.GA.T.CACC
	TRBJ3-3*01	Jβ3	T.CTG.AA.TACGCTCTAT
	TRBJ4-4*01	Jβ4	TT.CCA..GAAAGAT.A
	TRBJ5-1*02	Jβ5	TAA.AACC.GGCTCCGC.T
	TRBJ6-1*01	Jβ6	TTC…TAAT.CGCC.CTCTA.

*Sequences are shown in comparison to a representative gene segment. The strain of origin is indicated.*

### Cloning of Representative CDR3 Sequences From Pre Selection Thymocytes

To assess the effect of the surrounding locus on natural selection of CDR-B3 and identify how the elements that contribute to CDR3 are being processed during development, we compared CDR-B3 content in DβYTL DN2 thymocytes to a panel of bone marrow proB and thymocyte DN2 controls. Most of our previous studies of the CDR-H3 repertoire were performed in BALB/c mice, hence sequences from BALB/c bone marrow pro B cells were used as a major control for how DSP gene segments are normally handled. However, since the DβYTL allele was studied in C57BL/6 mice, we also compared CDR-H3 sequences from wild type C57BL/6 bone marrow proB cells as an additional control. We controlled for the effect of deleting the Dβ2-Jβ2 gene segment cluster by analyzing CDR-B3 sequences from Dβ2ko DN2 thymocytes, where the wild type Dβ1-Jβ1 locus was intact and the Dβ2-Jβ2 locus had been deleted. Together with the CDR-H3 sequences, study of these CDR-B3 sequences allowed comparison of how DSP gene segments were handled in two different strains and in the context of the TCR locus, as well as between the single DSP gene segment and Dβ1, both in the presence and absence of the Dβ2-Jβ2 locus.

We had previously cloned and sequenced immunoglobulin HC transcripts from BALB/c and C57BL/6 Fraction B cells that used members of the V_*H*_7183 family (4, 9). From this library of sequences, we identified 96 DSP containing sequences from BALB/c and 24 from C57BL/6 (Figure 1). To sample how the elements that contribute to CDR-B3 content are being processed, we randomly chose to analyze CDR-B3 sequences from TCR Vβ13.1 containing transcripts. We PCR amplified TCRβ sequences containing Vβ13.1 from DN2 thymocytes from DβYTL, Dβ2ko and wild type C57BL/6 mice. We identified 47, 47 and 29 CDR-B3 sequences (Figure 1), respectively. Among the sequences obtained from the wild type mice, we excluded those in which the Dβ1 gene segment had rearranged to a Jβ2 gene segment.

**FIGURE 2:**
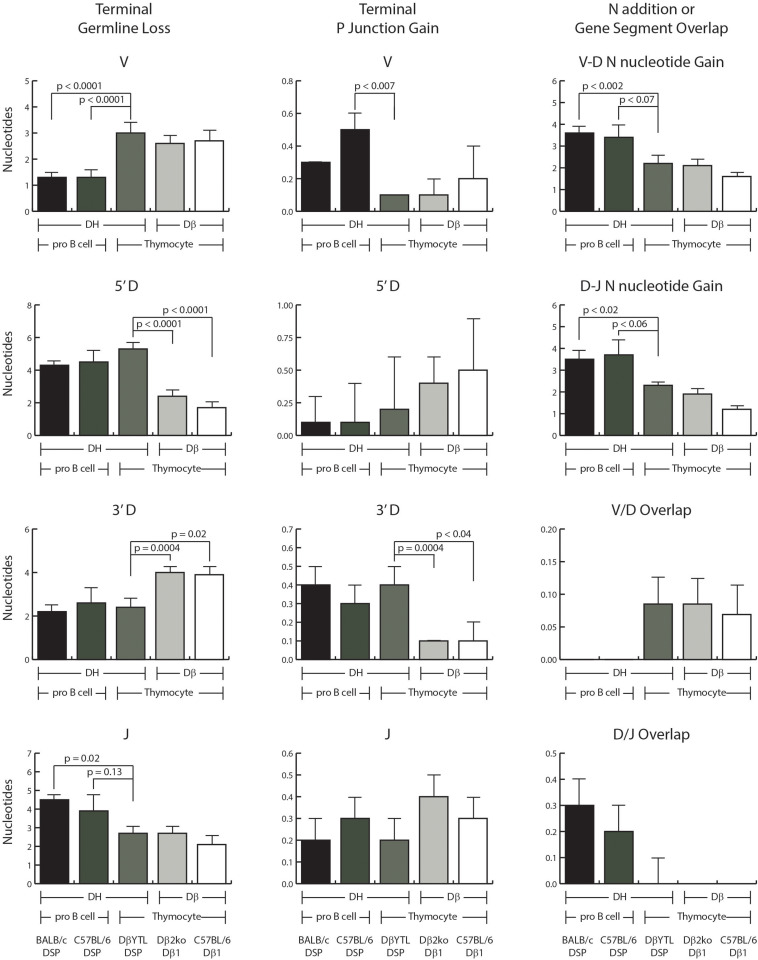
Comparisons of VDJ processing in the pre-selected repertoire. Shown in the **left column** is the average loss of terminal nucleotides in the V, D, and J gene segments. Shown in the **middle column** is the average amount of P junction addition. Shown in the **right column** is: row 1 and 2, the average amount of N nucleotide addition between V and D and between D and J, respectively; row 3 and 4, the extent of V/D and D/J overlap in sequence among sequences lacking N addition. In each case, the data are ordered, from left to right: wild type BALB/c DSP containing sequences, wild type C57BL/6 DSP containing sequences, DβYTL containing sequences, Dβ1 containing sequences from the Dβ2ko mice and from wild type C57BL/6 mice. From left to right, the first three columns represent D_*H*_ sequences and columns four and five are Dβ sequences. From left to right, the first two columns of each graph represent data from Fraction B proB cells, followed by the three columns of each graph representing data from DN2 cells from thymocytes. Error bars display the standard error of the mean. Statistical analysis was performed only for DβYTL in comparison with the four controls, and comparisons lacking a “*p*” value all have *p* > 0.05.

**FIGURE 3:**
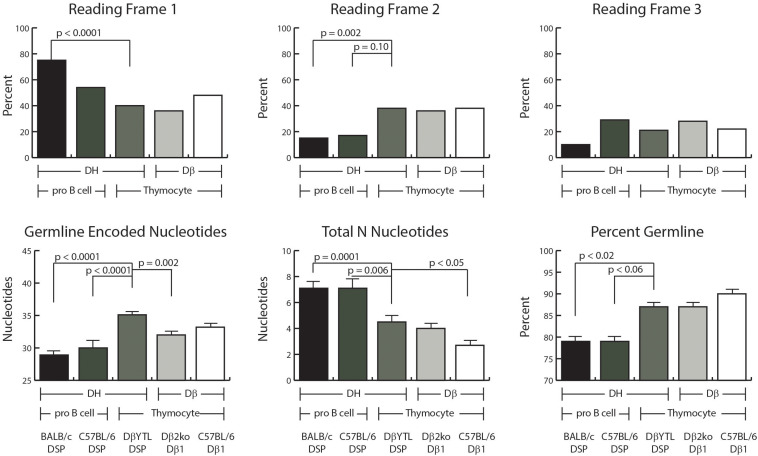
D reading frame usage and the relative distribution of germline encoded nucleotides and N addition. **Top row:** the percent of sequences using the indicated reading frame. **Bottom row:** the average number of germline encoded nucleotides in the CDR3 interval **(left)**, the average number of total N nucleotides in the CDR3 interval **(middle)**, and the average percent of germline encoded nucleotides in each CDR3 **(right)**. From left to right, the first two columns of each graph represent data from Fraction B lineage cells, followed by the three columns of each graph representing data from thymocytes. Error bars display the standard error of the mean. Statistical analysis was performed only for DβYTL in comparison with the four controls, and comparisons lacking a “*p*” value all have *p-*values of >0.05.

**FIGURE 4:**
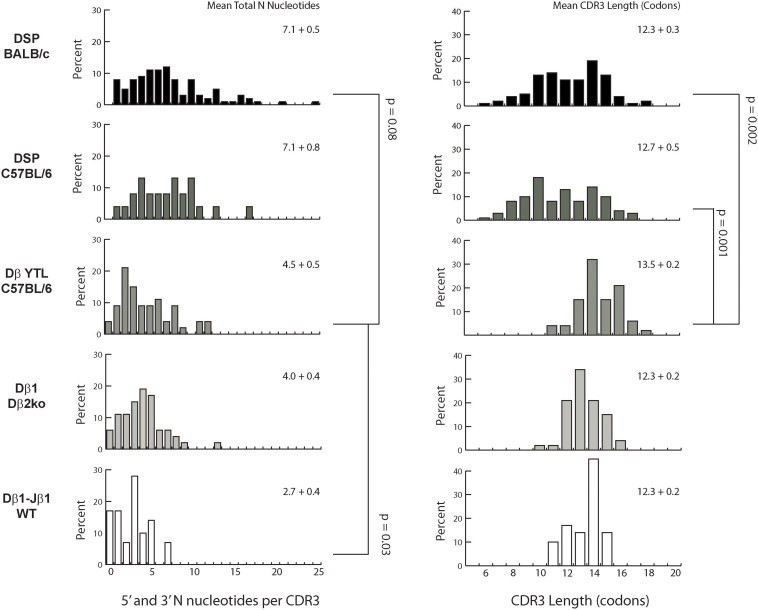
Distribution of total N addition in nucleotides and CDR3 length in codons. **Left column:** percent of CDR sequences that contain between 0 and 25 N nucleotides. From top to bottom: Fraction B BABL/c and pro B C57BL/6 CDR-H3 sequences, followed by CDR-B3 sequences from DβYTL, Dβ2ko, and Dβ1 wild type thymocytes. Statistical analysis was performed only for DβYTL in comparison with the four controls, and comparisons lacking a “*p*” value all have *p* values of >0.05. Means for the total number of N nucleotides **(left column)** and for CDR3 length **(right column)** are given in the **upper right** of each panel.

**FIGURE 5:**
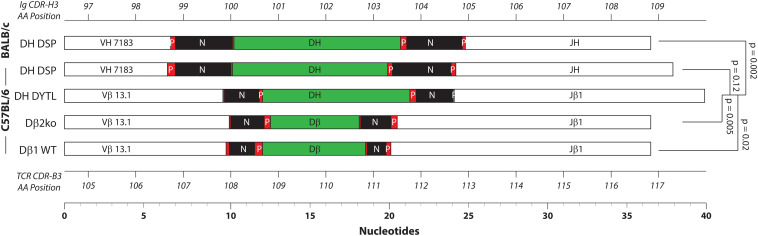
Relative distribution of the CDR3 components. Shown together are the average length of V, D, and J gene segments, P junctions, and N addition in nucleotides for each of the mouse strains. From top to bottom: Fraction B BABL/c and pro B C57BL/6 CDR-H3 sequences, followed by CDR-B3 sequences from DβYTL, Dβ2ko, and Dβ1 wild type thymocytes. Statistical analysis was performed only for DβYTL in comparison with the four controls. Shown at the **top** is the codon position number for CDR-H3, and at the **bottom** the codon position number for CDR-B3.

In Figures 2–5, we display the data in the order from left to right or from top to bottom for sequences obtained from BALB/c wild type Fraction B proB cells, C57BL/6 wild type Fraction B pro B cells, C57BL/6 DβYTL DN2 thymocytes, C57BL/6 Dβ2ko DN2 thymocytes, and C57BL/6 wild type DN2 thymocytes. This arrangement facilitates visual comparison of the effects of D sequence and cell lineage on VDJ recombination and N region addition. Statistical comparisons are only shown for DβYTL versus the other four mouse strains. Comparisons where the *p* value is not shown had a *p* > 0.05.

### Loss of Terminal D Nucleotides Is D Sequence Specific

There was a greater loss of nucleotides at the 3′ end of the V and a lesser loss of nucleotides at the 5′ end of the J in DN2 thymocytes than in Fraction B proB cells, irrespective of the sequence of the D (Figure 2). Conversely, the loss of 5′ D sequence was greater and the loss of 3′ D sequence lesser in progenitor cells that contained a DSP sequence than in progenitor cells that contained a Dβ1 sequence irrespective of the host cell type. Thus, although the sequence of the D did not control V or J nucleotide loss; the sequence of the D did control terminal loss of D nucleotides, irrespective of cell type. This is further evidence that the sequence of the D controls how that sequence is modified during VDJ rearrangement, with each D creating its own D-specific repertoire (8).

P junction gain at the termini of the D’s also appeared D gene segment-specific. There was a greater gain of P junctions at the 5′ end of the Dβ sequence than the DSP sequence, and a greater gain of P junction sequence at the 3′ end of DSP sequences than at the 3′ end of the Dβ sequence, irrespective of cell type, although the differences at the 5′ end did not achieve statistical significance. There was a greater gain of P junction nucleotides at the 3′ terminus of V gene segments in Fraction B cells than in thymocytes irrespective of the sequence of the D. The differences in P junctions in J’s were not statistically significant.

V/D overlap was greater in thymocytes and D/J overlap greater in Fraction B proB cells, although again these differences did not achieve statistical significance and the absolute contribution of nucleotides was small.

The presence of two or more microhomologous nucleotides between rearranging gene segments has been shown to influence the site of RAG mediated recombination in progenitor B cells (12, 13). Neither the 5′ terminus of the DSP gene segments nor the Dβ1 segment share dinucleotide microhomology with the 3′ terminus of the Ig or TCR V’s. The 3′ terminus of all DSP gene segments shares at least a two nucleotide microhomology (AC) with the 5′ sequences of J_*H*_1, J_*H*_2, and J_*H*_4. There is only a two nucleotide microhomology between DSP gene segments and Jβ1 and Jβ2. There is no shared two or more nucleotide microhomology between the other four Jβ’s and the 3′ terminus of DSP. The 3′ terminus of Dβ1 ends in GC, a dinucleotide that is not found at the 5′ terminus of any of the Jβ1 gene segments. The lack of a detectable effect of changing the sequence of the D on the extent of terminal loss of V or J sequences and the preservation of patterns of D nucleotide loss irrespective of the cell types supports the view that it is the germline sequence of the V, D, and J gene segments that auto-regulates terminal P junction gain or exonucleolytic loss of sequence at the time of gene segment recombination.

### The Extent of N Region Addition Is Cell Type Specific

The extent of N region addition between V and D as well as between D and J was greater in Fraction B proB cells than in DN2 thymocytes, irrespective of the sequence of the D (Figure 2).

### Reading Frame Usage Is Random in DN2 Thymocytes Irrespective of D Sequence

Partly due to the microhomology between the 3′ end of the D and the 5′ end of the J, developing B cells demonstrate a bias against rearrangement into reading frame 2 (RF2). In the absence of extensive DβYTL-Jβ microhomology, RF 2 usage increased to a third of the rearrangements (Figure 3). Enrichment for RF1 was greater in BALB/c than in C57BL/6 with a compensatory loss of RF3. The mechanism underlying this difference is unclear.

### On Average, CDR-H3 Contains More Random N Nucleotides and Exhibits Greater Variance in Sequence Than CDR-B3

The average number of total N nucleotides (5′ plus 3′) was greater in progenitor B cells than in thymocytes (Figure 3). Although there was no statistically significant difference in total N nucleotides between DβYTL CDR-B3 than in Dβ1-Jβ1 sequences from Dβ2ko, there was less N addition in wild type Dβ1-Jβ1 sequences. The trend to have less N addition was observed both at the V→DJ and D→J joins. How the presence of an additional DβJβ locus might influence total N addition is unclear.

Differences in N addition between cell types were also found in the variance and distribution of N nucleotides (Figure 4). The most variable range of lengths was observed in CDR-H3 of BALB/c transcripts and the least in CDR-B3 from wild type thymocyte DN2-derived TCRβ transcripts. Thus, the cell type clearly influenced the extent of N addition. There is also the suggestion that the absence of Dβ2-Jβ2 not only affected the absolute number of N nucleotides added, but also influenced the variance of N nucleotide addition.

The greater variance of N addition in fraction B proB cells vs. DN2 thymocytes contributed to a marked difference in the variance of the number of amino acids, or the lengths of the sequences, in CDR-H3 versus CDR-B3 (Figure 4). The variance in lengths was greater in progenitor B cells than in thymocytes. In DβYTL, the variance in lengths was similar to that of Dβ2ko and wild type CDR-B3. Due in part to the difference in both the quantity and variability of N addition, the variance in the distribution of CDR-B3 lengths was significantly lower than the variance in the distribution of CDR-H3 lengths.

When viewed *in toto*, the greater length of the Vβ and Jβ portions of CDR-B3 coupled with the lower amount of N addition results in the average CDR-B3 containing more germline-encoded sequence than CDR-H3 (Figure 5). Intriguingly, however, despite differences in N addition and terminal nucleotide loss or gain, the average length of immunoglobulin CDR-H3s containing a DSP gene segment proved similar to that of TCR CDR-B3 containing Dβ1 and Jβ1 gene segments. This balance was affected when the sequence of the D_*H*_ was lengthened. DSP2.3 is five nucleotides longer than Dβ1, and on average the length of CDR-B3 sequences containing DSP2.3 was 3.5 nucleotides longer than those containing Dβ1 gene segment sequence. Thus the length of CDR-B3 can be heavily influenced by the length and terminal sequence of the D.

## Discussion

Adaptive immunity in jawed vertebrates is designed to produce immunoglobulin (Ig) and T cell receptor (TCR) repertoires of astronomic diversity in developing B cells and thymocytes (1, 2). These two antigen receptors have overlapping but distinct roles. While both Ig and TCR act as the antigen receptor for their respective host cells, Igs also have effector functions that require high affinity binding of high specificity. Conversely, while the interaction between Ig and antigen is bimolecular, T cell receptor recognition of antigen requires a trimolecular interaction with both peptide antigen and a member of the major histocompatibility complex (MHC). Secreted Ig clears pathogens through binding to target antigens, which induces a cascade of humoral and cellular reactions. On the other hand, T cells via their TCR can induce killing of target cells infected with the pathogens. Based on these major differences in functions, it is not surprising that the measures used to control their repertoires vary, even though genes encoding both receptors undergo the same process of VDJ recombination and N addition.

We have previously focused our efforts on testing whether the functional efficiency of the process of immunoglobulin diversification can be enhanced by means of natural selection of germline sequence (4). In addition to immunoglobulin, the diversity provided by VDJ recombination and N addition enables a broad array of TCR antigen specificity. The antigen receptors that are generated by both the immunoglobulin and TCR loci have the capacity to be autoreactive, protective, superfluous, or ineffective. Thus a completely random process would likely result in costly inefficiency. We have previously shown that the sequence of D_*H*_ can heavily influence immunoglobulin diversity, and that the features of this influence can be detected in broad outline by analysis of as few as ten to twenty sequences. Moreover, the changes induced by altering D_*H*_ sequence negatively impact B cell development, antibody production, protection against infection, responses to allergens, and susceptibility to autoreactive antibody production (4, 14–16).

In this work, we sought to test whether the process of VDJ recombination and N region addition would be altered in T cells should the sequence of Dβ be changed. To test for potential germline constraints on both Ig and TCR repertoires, we replaced Dβ1 with a commonly used D_*H*_ in a mouse lacking the Dβ2-Jβ2 gene segment cluster. We evaluated the criteria of selection of TCR which included terminal nucleotide loss, terminal nucleotide P junction gain, V/D and D/J overlap, N addition, reading frame use, the relative contribution of N addition versus germline encoded content in CDR3, and total CDR3 length.

We compared the repertoires expressed in proB cells from the bone marrow to DN2 cells from the thymus. In developing B cells, D_*H*_→J_*H*_ rearrangement precedes V_*H*_→D_*H*_J_*H*_ rearrangement. Hardy Fraction B progenitor B cells contain these initial VDJ joins and by definition do not express appreciable levels of μH chain protein. Thus, the VDJ repertoire they express is considered to be preimmune because it is unselected by the immune properties of μH chain protein, including preBCR formation and antigen binding. Similarly, in the thymus Dβ→Jβ rearrangement precedes Vβ→DJβ rearrangement and, during thymocyte development, VDJCβ transcripts are typically first found in DN2 cells. The homology between proB and DN2 cells is not exact since the definition of DN2 cells does not depend on the presence or absence of TCRβ protein. While it is possible that expression of nascent TCRβ protein could affect the fate of the cell, either directly or through its interaction with preTCRα, selection of the TCR β chain is primarily associated with the developmental checkpoint between DN3a and DN3bc (17). Thus, as with proB cells, DN2 thymocytes are considered to primarily express a preimmune TCRβ repertoire.

We found that the sequence of the D maintains control over the outcome of the rearrangement of the D regardless of whether recombination is occurring in proB cells or thymocytes. While the sequence of the D has minimal effects on V or J gene segment loss or gain of terminal sequence, it self-directs the extent of terminal D nucleotide loss or P junction gain.

The extent and variance in N addition appears affected by cell type and by the loci surrounding the rearranging gene segments (18, 19). This study was not designed to fully evaluate the independent contributions of these factors. However, it did disclose a potential role for the Dβ2-Jβ2 locus in regulating the extent and distribution of N nucleotide addition. The activity of the N region addition machinery is regulated during ontogeny by controlling TdT expression and access to exposed terminal DNA sequence (20–22). We would surmise that one or both of these mechanisms are the means by which cell type and the presence or absence of a rearranging locus could influence N region length (20).

Structural studies have shown that amino acids at the tip of the TCR CDR-B3 loop are highly likely to interact directly with peptide antigens presented on the surface of the MHC molecule. The length distribution of CDR-B3 has been proposed to be restricted largely by MHC-specific selection, disfavoring TCRs with CDR3s that are longer than 13 amino acids (23). Our data would suggest that initial restrictions are controlled by natural selection. Potential mechanisms include preservation of D region sequence and regulation of N addition (24).

On average, the 3′ termini of TCR Vβ and the 5′ termini of TCR Jβ are longer than their immunoglobulin V_*H*_ and J_*H*_ counterparts (e.g., Table 1 and Supplementary Table S2). Natural selection of the germline sequences of TCR Vβ, Dβ, and Jβ coupled with control of CDR-B3 length variance has the effect of focusing the diversity provided by N addition and the sequence of the D to that portion of CDR-B3 that is most likely to interact with the peptide that is bound to the presenting MHC.

This manuscript has focused on the effect of D sequence on the preimmune repertoire at the nucleotide level. The contribution of the amino acids encoded by the D, which differ in peptide signature between T cells and B cells, to repertoire selection, T cell development, and antigen responses is reported in a companion manuscript. Given our past experience with the immunoglobulin locus, we were not surprised that T cell biology is also heavily affected by violation of normal germline constraints on the amino acids encoded by the sequence of the D. We speculate that the threat of unrestrained inefficiency or potential hazard in the creation of antigen receptor repertoires by an entirely stochastic process of DNA rearrangement appears to have been constrained during evolution by controlling the sequences of the rearranging gene segments to optimize the products of recombination while engendering diversity.

## Data Availability Statement

The authors acknowledge that the data presented in this study must be deposited and made publicly available in an acceptable repository, prior to publication. Frontiers cannot accept a manuscript that does not adhere to our open data policies.

## Ethics Statement

The animal study was reviewed and approved by UAB IACUC.

## Author Contributions

MK took the lead in analyzing and interpreting the data, and writing the manuscript. ML took the lead role in sequencing of the TCR transcripts from thymocytes. RS participated in planning the original studies, creating the mice, and performing the initial analysis of T cell and repertoire development. PK was instrumental in the creation of the mice. PB participated in the planning of the experiments, interpreting the data, and editing the manuscript. HS developed the concept of the project, directed the planning and execution of the studies, reviewed the data, and directed the writing of the manuscript. All authors contributed to the article and approved the submitted version.

## Conflict of Interest

The authors declare that the research was conducted in the absence of any commercial or financial relationships that could be construed as a potential conflict of interest.

## Supplementary Material

FIGURE S1 Creation of the DβYTL targeting construct.

FIGURE S2 Flow scheme for analysis and sorting of T cell subsets in the D altered and WT mice. DN thymocytes gated on live, lineage negative (CD3, CD4, CD8, CD11b, B220, and NK1.1) singlets. DN2 thymocytes gated on CD25 and CD44.

TABLE S1 TCR CDR-B3 sequences.

TABLE S2 Germline V, D, and J CDR3 contributing sequences pertinent to this study.
