# Supplementary figures and images for: Preimmune Control of the Variance of TCR CDR-B3: Insights Gained From Germline Replacement of a TCR Dβ Gene Segment With an Ig DH Gene Segment
Source: Front Immunol. 2020 Sep 11;11:2079. doi: 10.3389/fimmu.2020.02079 (PMC7518465; doi:10.3389/fimmu.2020.02079)

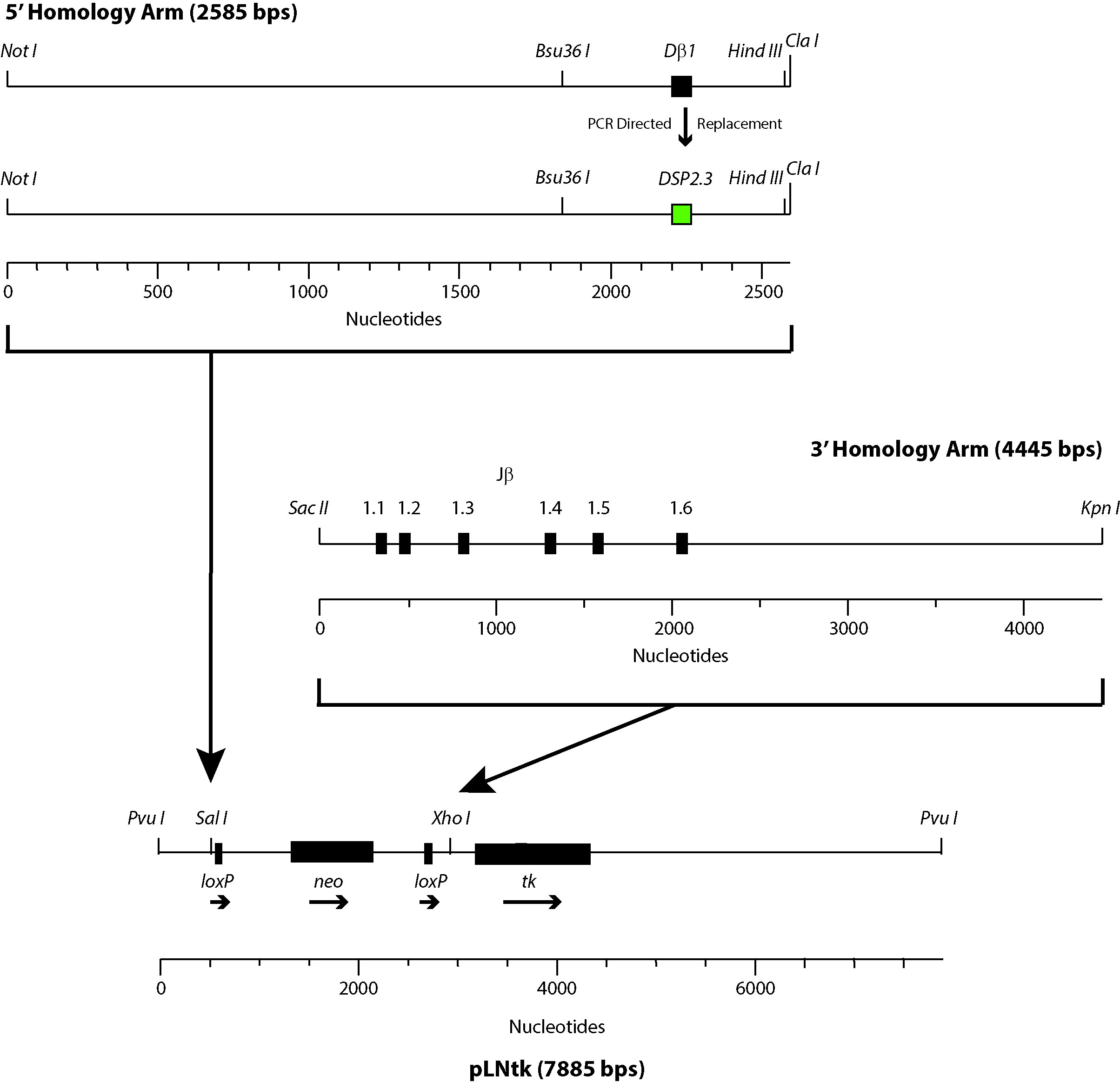

Supplement: FIGURE S1 — Creation of the DβYTL targeting construct. [file Image_1.JPEG]

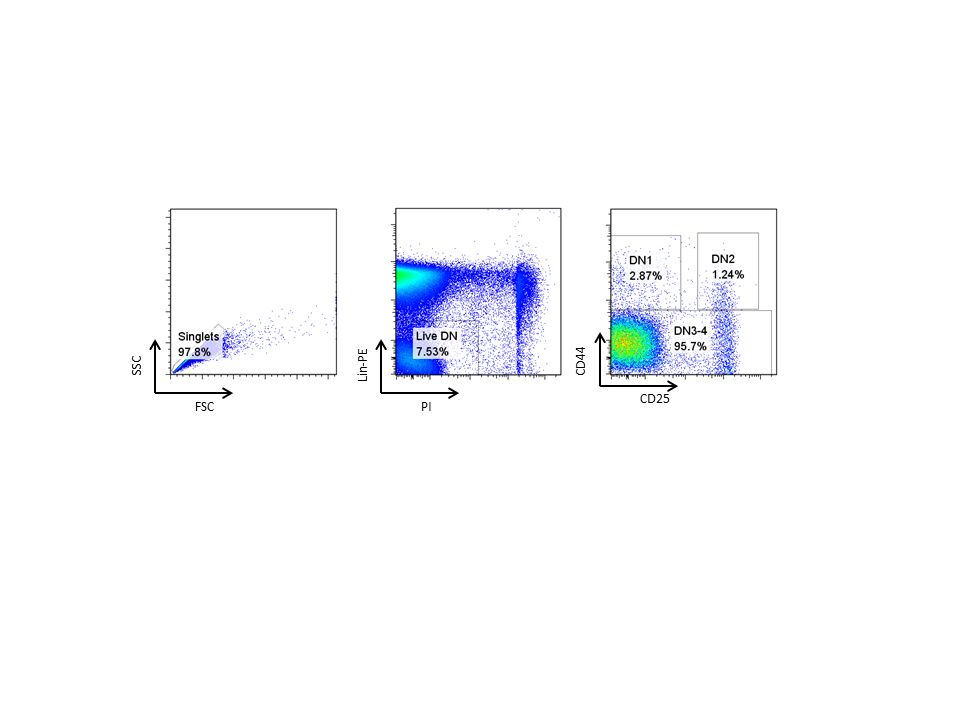

Supplement: FIGURE S2 — Flow scheme for analysis and sorting of T cell subsets in the D altered and WT mice. DN thymocytes gated on live, lineage negative (CD3, CD4, CD8, CD11b, B220, and NK1.1) singlets. DN2 thymocytes gated on CD25 and CD44. [file Image_2.png]
