# Supplementary material for: Preimmune Control of the Variance of TCR CDR-B3: Insights Gained From Germline Replacement of a TCR Dβ Gene Segment With an Ig DH Gene Segment
Source: Front Immunol. 2020 Sep 11;11:2079. doi: 10.3389/fimmu.2020.02079 (PMC7518465; doi:10.3389/fimmu.2020.02079)
Supplement: TABLE S2 — Germline V, D, and J CDR3 contributing sequences pertinent to this study. [file Table_2.docx]

**Supplemental Table 2**. Germline V, D and J CDR3 contributing sequences pertinent to this study.

**Strain IMGT Common 3’ Terminus**

**BALB/c IGHV5-01 VH81X GCAAGACA**

**IGHV5-02 VHD6.96 GCAAGAGA**

**IGHV5-06 VH3:3.39 GCAAGACA**

**IGHV5-14 7183.14 GCAAGACA**

**IGHV5-05 VH50.1 GCAAGACA**

**IGHV5-09 7183.9 GCAAGACA**

**IGHV5-04 VH37.1 GCAAGACA**

**7183.9T GCAAGACA**

**IGHV5-16 98-3G GCAAGGGA**

**IGHV5-03 VH283 GCAAGATA**

**IGHV5-10 7183.1O GCAAGACA**

**IGHV5-08 VH10-19 ACAAGAGA**

**IGHV5-15 68-5N GCAAGAGA**

**IGHV5-17 69-1 ACAAGAGA**

**IGHV5-13 7183.13 GCAAGACA**

**IGHV5-12 57-1M GCAAGAGA**

**IGHV5-11 VH7183.11 GCAAGGGA**

**C57BL/6 IGHV5-17 GCAAGG**

**IGHV5-16 GCAAGAGA**

**IGHV5-15 GCAAGACA**

**IGHV5-14 GCAAGACA**

**IGHV5-12 GCAAGACA**

**IGHV5-09 GCAAGACA**

**IGHV5-06 GCAAGACA**

**IGHV5-04 GCAAGAGA**

**IGHV5-02 GCAAGACA**

**TRBV13-1 GCCAGCAGTGATG**

**Strain IMGT Common D sequence**

**IGHD2-02*01 DSP2.03 TCTACTATGGTTACGAC**

**IGHD2-04*01 DSP2.02 TCTACTATGATTACGAC**

**IGHD2-02*01 DSP2.04 TCTACTATGGTTACGAC**

**IGHD2-01*01 DSP2.05 TCTACTATGGTAACTAC**

**IGHD2-10*01 DSP2.07 CCTACTATGGTAACTAC**

**IGHD2-11*01 DSP2.08 CCTAGTATGGTAACTAC**

**IGHD2-03*01 DSP2.09A TCTATGATGGTTACTAC**

**IGHD2-14*01 DSP2.11 CCTACTATAGGTACGAC**

**C57BL/6 IGHD2-03*01 DSP2.09A TCTATGATGGTTACTAC**

**IGHD2-04*01 DSP2.02 TCTACTATGATTACGAC**

**IGHD2-05*01 DSP2.x CCTACTATAGTAACTAC**

**IGHD2-06*01 DSP2.x CCTACTATAGTAACTAC**

**IGHD2-07*01 DSP2.03 TCTACTATGGTTACGAC**

**IGHD2-08*01 DSP2.05 TCTACTATGGTAACTAC**

**TRDB1*01 Dβ1 GGGACAGGGGGC**

**Strain IMGT Common 5’ Terminus**

**BALB/c IGHJ1*01 JH1 CTACTGGTACTTCGATGTC**

**IGHJ2*01 JH2 ACTACTTTGACTAC**

**IGHJ3*01 JH3 CCTGGTTTGCTTAC**

**IGHJ4*01 JH4 ATTACTATGCTATGGACTAC**

**C57BL/6 IGHJ1*03 JH1 CTACTGGTACTTCGATGTC**

**IGHJ2*01 JH2 ACTACTTTGACTAC**

**IGHJ3*01 JH3 CCTGGTTTGCTTAC**

**IGHJ4*01 JH4 ATTACTATGCTATGGACTAC**

**TRBJ1-1*01 Jβ1 CAAACACAGAAGTCTTC**

**TRBJ2-2*01 Jβ2 CAAACTCCGACTACACC**

**TRBJ3-3*01 Jβ3 TTCTGGAAATACGCTCTAT**

**TRBJ4-4*01 Jβ4 TTTCCAACGAAAGATTA**

**TRBJ5-1*02 Jβ5 TAACAACCAGGCTCCGCTT**

**TRBJ6-1*01 Jβ6 TTCCTATAATTCGCCCCTCTAC**
